# Differential Association of Cx37 and Cx40 Genetic Variants in Atrial Fibrillation with and without Underlying Structural Heart Disease

**DOI:** 10.3390/ijms19010295

**Published:** 2018-01-19

**Authors:** Sebastian Carballo, Anna Pfenniger, David Carballo, Nicolas Garin, Richard W James, François Mach, Dipen Shah, Brenda R Kwak

**Affiliations:** 1 Service of General Internal medicine, University Hospitals of Geneva, 1211 Geneva, Switzerland; nicolas.garin@hcuge.ch; 2 Department of Pathology and Immunology, University of Geneva, 1211 Geneva, Switzerland; anna.pfenniger@gmail.com (A.P.); Brenda.KwakChanson@unige.ch (B.R.K.); 3 Service of Cardiology, University Hospitals of Geneva, 1211 Geneva, Switzerland; david.carballo@hcuge.ch (D.C.); francois.mach@hcuge.ch (F.M.); dipen.shah@hcuge.ch (D.S.); 4 Service of Endocrinology and Diabetes, University Hospitals of Geneva, 1211 Geneva, Switzerland; richard.james@hcuge.ch

**Keywords:** atrial fibrillation, connexin, polymorphism, genetic variant

## Abstract

Atrial fibrillation (AF) appears in the presence or absence of structural heart disease. The majority of foci causing AF are located near the ostia of pulmonary veins (PVs), where cardiomyocytes and vascular smooth muscle cells interdigitate. Connexins (Cx) form gap junction channels and participate in action potential propagation. Genetic variants in genes encoding Cx40 and Cx37 affect their expression or function and may contribute to PV arrhythmogenicity. DNA was obtained from 196 patients with drug-resistant, symptomatic AF with and without structural heart disease, who were referred for percutaneous catheter ablation. Eighty-nine controls were matched for age, gender, hypertension, and BMI. Genotyping of the Cx40 −*44G* > *A*, Cx40 +*71A* > *G*, Cx40 −*26A* > *G*, and Cx37 *1019C* > *T* polymorphisms was performed. The promoter A Cx40 polymorphisms (−*44G* > *A* and +*71A* > *G*) showed no association with non-structural or structural AF. Distribution of the Cx40 promoter B polymorphism (−*26A* > *G*) was different in structural AF when compared to controls (*p* = 0.03). There was no significant difference with non-structural AF (*p* = 0.50). The distribution of the Cx37 *1019C* > *T* polymorphism was different in non-structural AF (*p* = 0.03) but not in structural AF (*p* = 0.08) when compared to controls. Our study describes for the first time an association of drug-resistant non-structural heart disease AF with the Cx37 *1019C* > *T* gene polymorphism. We also confirmed the association of the Cx40 − *26G* > *A* polymorphism in patients with AF and structural disease.

## 1. Introduction

Atrial fibrillation (AF) is the most common sustained cardiac arrhythmia, its incidence increasing with age to reach up to 35 new cases per 1000 population in the elderly [1,2]. In the general population, its prevalence is estimated at 1.5–2%. AF is associated with increased risk of stroke, incidence of heart failure, and an overall higher mortality [3,4]. Diagnosis of AF is based on electrocardiographic characteristics such as rapid, irregular, fibrillatory waves, varying in size, shape, and timing [1]. Five clinical types of AF are recognized, mainly based on duration [2,3,4,5]: paroxysmal AF, persistent AF, long-standing persistent AF, and permanent AF. Whilst controversial, the term idiopathic or lone AF is used in reference to AF appearing in the absence of structural disease such as atrial left enlargement or valvular heart disease [6]. This historical concept and term should probably and progressively be avoided as understanding of the multiple complex etiologies of AF is improved [7].

When underlying structural heart disease is present, such as in patients with hypertensive heart disease, valvulopathy, or ischemic heart disease, this may trigger progressive remodeling of both the ventricles and atria [3,4]. Structural abnormalities will induce remodeling of the myocardium by affecting the extracellular matrix and cardiomyocytes through inflammatory processes and increased fibrosis [3,4]. Electrophysiological alterations and remodeling are instrumental in maintaining AF after its onset [8]. Initiation of AF is thought to depend on a combination of abnormal impulse formation, conduction and a propensity for reentry at the ostia of pulmonary veins (PVs), where sheets of cardiomyocytes and vascular smooth muscle cells (VSMCs) interdigitate [9,10]. However, the molecular mechanism underlying this regional specificity remains to be discovered.

Variants in genes encoding for membrane channels have been associated with both familial and other forms of AF, including genes encoding potassium channels such as *KCNQ1* and *KCNA5* [11,12,13]. Interestingly, atrial vulnerability to AF has been associated with polymorphisms in the Connexin40 (Cx40) gene, *GJA5* [14,15,16,17]. In addition, specific somatic mutations in the same gene have been discovered in idiopathic AF [18]. Other clinical AF presentations have been described with mosaicism in another connexin gene, *GJA1* encoding for Cx43 [19].

Connexins are gap junction proteins playing an essential role in direct cell–cell communication in the vast majority of tissues in the body, including in electrical propagation in the heart [20]. There are 20 different connexins in humans, each forming channels with distinct properties and with specific expression patterns [21]. Cx40, encoded by *GJA5*, is expressed by endothelial cells (ECs), by coronary VSMCs, by atrial cardiomyocytes, and in the cardiac conduction system. Cx37, encoded by *GJA4*, is found in ECs, pulmonary vein VSMCs, monocyte/macrophages, and platelets. Cx43, encoded by *GJA1*, is expressed by ventricular and atrial cardiomyocytes, VSMCs, ECs, and monocyte/macrophages [22]. Several polymorphisms have been described in *GJA4* and *GJA5*, which affect either their connexin expression level or gap junction channel function, some of which may contribute to the arrhythmogenicity of the PVs [15]. In animal studies, downregulation of either Cx40 or Cx43 expression increases susceptibility to AF [23,24].

Variants in the Cx37 (*GJA4*) gene have been associated with atherosclerosis and coronary heart disease, most likely through their influence on monocyte adhesion, thereby regulating the extent of local inflammation [25]. Both systemic and local inflammation appear to influence the development of AF in various situations, in patients having undergone cardiac surgery as well as non-post-operative patients [26].

Here, we investigated the relation between polymorphisms in *GJA5* and *GJA4* in defined AF populations. Within the AF population, a distinction was made between patients with no underlying structural cardiomyopathy, for the purpose of clarity referred to as non-structural AF, and those with clearly documented structural disease, referred to as structural AF. All AF patients that were included had undergone a trial of anti-arrhythmic drugs and were referred for catheter ablation because of poor response.

## 2. Results

A total of 285 individuals were included in this study; 92 patients in the non-structural AF group, 104 patients with structural AF, and 89 controls. Characteristics of the study population are summarized in Table 1.

The type of cardiomyopathy present in the structural AF group is detailed in Table 2. Valvular disease and dilated atria were the main structural abnormalities.

### 2.1. Cx40 − 44G > A/+71A > G Polymorphisms

Analysis of the two promoter A Cx40 polymorphisms (−*44G* > *A* and +*71A* > *G*) showed an almost complete linkage disequilibrium of the −*44G*/+*71A* and −*44A*/+*71G* alleles. The overall genotype distribution in the control group was −*44GG*/+*71AA* = 67.4%, −*44AG*/+*71AG* = 28.1% and −*44AA*/+*71GG* = 4.5%. This genotype distribution was not significantly different in either of the AF groups (non-structural AF: *p* = 0.846; structural AF: *p* = 0.132) (Table 3). There was also no significant difference in between the two AF groups (*p* = 0.275).

### 2.2. Cx40 − 26G > A Polymorphism

The specific genotype distribution of the Cx40 − *26G* > *A* polymorphism in the control group was −*26GG* = 25.8%, −*26AG* = 47.2%, and −*26AA* = 27.0%. This distribution was significantly different in the structural AF group: −*26GG* = 26.0%, −*26AG* = 61.5%, and −*26AA* = 12.5% (*p* = 0.029). There was no significant difference between the control and the non-structural AF group (*p* = 0.511) (Table 3) or between the two AF groups (*p* = 0.3).

### 2.3. Cx37 1019C > T Polymorphism

The specific genotype distribution of the Cx37 *1019C* > *T* polymorphism in the control group was *1019CC* = 42.7%, *1019CT* = 34.8%, and *1019TT* = 22.5%. This distribution was significantly different in the non-structural AF group: *1019CC* = 46.7%, *1019CT* = 44.6%, and 1019TT = 8.7% (*p* = 0.034). There was no significant difference between the control and the structural AF group (*p* = 0.080) (Table 3) or between the two AF groups (*p* = 0.85).

## 3. Discussion

Our study describes for the first time an association of the Cx37 *1019C* > *T* gene polymorphism with drug-resistant non-structural AF, but not with structural AF. The potential differences in gene susceptibility between non-structural and structural AF were further underlined by the fact that we found an association of the Cx40 − *26G* > *A* polymorphism with structural AF and not with non-structural AF.

These results suggest that there are potential differences in the genetic susceptibility to AF in given populations. Patients who develop AF in whom there is no underlying structural cardiac disease appear to have an increased frequency of Cx37 *1019C* alleles than patients who do not develop AF. This genetic polymorphism has previously been described in populations with coronary heart disease and myocardial infarction [27,28]. The *GJA4* genotype may predict survival after an acute coronary syndrome [29]. Cx37 is principally expressed in endothelial cells, VSMCs, monocyte/macrophages, and platelets. The *1019C* > *T* polymorphism in *GJA4* causes a Proline-to-Serine substitution at amino acid 319 (P319S) in the cytoplasmic tail of Cx37, which in turn alters channel conductance and permeability [25,30]. The Cx37 polymorphism has been shown to affect monocyte adhesion as well as platelet aggregation [25,31]. Both mechanisms are thought to underlie the development of CAD [25]. Inflammation and its associated immune response are increasingly recognized to be involved in the initiation and maintenance of atrial fibrillation [32]. Thus, the Cx37 *1019C* > *T* polymorphism might affect the susceptibility to non-structural AF by virtue of its effect on monocyte adhesion. Alternatively, initiation of non-structural AF is thought to depend on a combination of abnormal impulse formation and conduction [9]. As Cx37 is also expressed in VSMCs at the ostia of pulmonary veins (PVs), where sheets of cardiomyocytes and VSMCs interdigitate, the differences in gap junction channel electrophysiological properties associated with the Cx37 *1019C* > *T* polymorphism may accentuate the latter mechanism [33].

Our results also show an association of the Cx40 − *26G* > *A* polymorphism in patients with AF with underlying cardiomyopathy, with the −26G allele being more frequent than in control patients. This single nucleotide polymorphism (SNP) in the promoter B region of *GJA5*, the *Cx40* gene, which alters the TATA box sequence and modulates mRNA expression levels of Cx40, has previously been associated with AF [34]. Of note, our study did not show any association of AF (either structural or non-structural) with the promoter A polymorphisms, Cx40 − *44*/+*71*; these polymorphisms were also shown to reduce *GJA5* expression in vitro by about 50%; however, Wirka et al. showed no effect on *GJA5* expression in atrial tissue, suggesting that promoter A is not necessary to Cx40 expression in atrial myocytes [34]. In patients with structural AF, atrial remodeling is often present at a structural, electrophysiological, and molecular level with changes in the refractory period and cardiomyocyte contractility [8,10]. Some of these atrial cardiomyocyte characteristics can be explained by changes in potassium channel properties and important changes to calcium handling [8]. Inconsistent observations have been made with respect to Cx40 expression levels in patients with AF, with both higher and lower levels being reported [35,36,37]. However, only few patients were included in these studies—10 and 12, respectively—and almost exclusively with mitral valve disease. Complete deletion of Cx40 in murine models leads to decreased atrial conduction velocity and increased atrial stability [8,38]. Somatic mutations in *GJA5* have been shown to predispose patients to idiopathic AF by either impairing the assembly of Cx40 into gap junctions or by affecting the electrical coupling itself [14,18]. Importantly, a recent study revealed that one of the reported Cx40 mutations (A96S) caused decreased atrial conduction velocities and sustained episodes of induced atrial fibrillation in mice and rat [39,40].

The strength of our study is the distinction between patients with or without underlying cardiomyopathy. This enables a more precise association study and a dissection of possible different pathophysiological mechanism leading to AF. We chose to include patients in whom anti-arrhythmic treatment was not successful in order to have long-standing or permanent AF. It may be interesting in the future to include all AF. The main limitation of our study is nevertheless the relatively small sample size, which increases the risk of falsely positive results. Furthermore, we acknowledge that non-structural AF is a misnomer and an exhaustive search of underlying etiology reduces the number of instances when there is truly no explanation for AF [7]. We also acknowledge that the link between the severity of valvular disease, in particular mitral valve disease, and AF may be complex. This may be an important aspect to further investigate. Associations between smoking or serum lipid levels and Cx40 have never been reported; it is, however, quite unlikely that one of these confounding factors would induce a significant association of the Cx40 − *26G* > *A* polymorphism with structural AF.

## 4. Materials and Methods

### 4.1. Patients

Three patient cohorts were studied, namely patients with AF and no underlying structural cardiac disease (non-structural AF), patients with AF and documented structural cardiac disease (structural AF), and a control population with no AF or underlying cardiac disease. This study protocol conforms to the ethical guidelines of the 1975 Declaration of Helsinki, as reflected in prior approval by the institution’s human research committee, namely the Internal Medicine Departmental Ethics Committee, Geneva University Hospital (Protocol 03-167, 3 April 2010). Informed consent was obtained from all patients. 

The non-structural AF group consisted of patients undergoing catheter ablation of AF by radiofrequency ablation through pulmonary vein isolation (RFAPVI). Inclusion criteria were an age > 18 years, documented paroxysmal or persistent AF (for <2 years) with or without typical flutter (ECG or Holter) after failure of at least one anti-arrhythmic treatment of Class I or III, with planned and clinically indicated RFAPVI. Exclusion criteria were reversible AF, clinical heart failure, increased left atrial size (>20 cm^2^ four chamber view), decreased left ventricular ejection fraction, any valvulopathy, or a history of prior AF or flutter ablation. The structural AF group consisted of patients undergoing RFAPVI but in whom there was underlying cardiomyopathy, be it structural or valvular disease, or documented ischemic heart disease (history of acute or chronic coronary disease, angiographically proven disease). Inclusion criteria were an age > 18 years, documented paroxysmal or persistent AF (for <2 years) with or without typical flutter (ECG or Holter) and with planned and clinically indicated RFAPVI. Inclusion and exclusion criteria for these groups are detailed in Table 4. Demographics and characteristics of each group are detailed in Table 1. The type of cardiomyopathy present in the structural AF group is detailed in Table 1. The control group was considered as a whole and variables were matched to the AF groups with priority given to age, gender, hypertension, and BMI, these being major risk factors associated with AF development. These individuals were from the same population basin as AF patients, seen in the same clinic, and were undergoing elective coronary angiography at the Geneva University Hospital due to symptoms of suspected CAD or unrelated conditions requiring angiographic evaluation [41]. Participants gave written informed consent for a blood draw at the time of angiography for use in confidential studies approved by the hospital’s institutional review board. Exclusion criteria for this control group were AF or a history of AF, or arrhythmia on 12 lead ECG, a history of coronary heart disease, any significant angiographic lesions on coronary angiography, or any known heart disease including valvulopathy.

### 4.2. Echocardiography

Echocardiography was performed on a HP/Philips 5500, Philips Electronics North American Corporation, Andover, MA, USA, with digitalization of images and analyzed according to a standardized protocol in a core lab.

### 4.3. DNA Extraction and Genotyping

DNA was extracted from the peripheral blood and genotyping of the Cx40 − *44G* > *A*, Cx40 + *71A* > *G*, Cx40 − *26A* > *G*, and Cx37 *1019C* > *T* polymorphisms was performed by polymerase chain reaction (PCR) and restriction fragment length polymorphism (RFLP) assays using previously established methods [34,42,43].

### 4.4. Statistical Analysis

Clinical, biological, and echocardiographic characteristics are presented using descriptive statistics and a number of cases with a percentage in parentheses for categorical variables. In view of skewed allele distribution and small numbers of certain genotypes, we used non-parametric tests. Proportions/categorical data were compared with the Fisher’s exact test.

## 5. Conclusions

Our study suggests that there may be a different genetic predisposition to non-structural AF as compared to AF appearing in the context of underlying heart disease. We report here for the first time that the Cx37 *1019C* > *T* variant is associated with non-structural AF. Mechanistically, this might involve effects of altered Cx37 channel function on the inflammatory response (monocyte adhesion) or on abnormal impulse conduction (communication between VSMCs and atrial cardiomyocytes). In addition, we confirmed that the Cx40 − *26G* > *A* polymorphism affecting the expression level of this protein in atrial cardiomyocytes is associated with structural AF.

## Figures and Tables

**Table 1:** Characteristics and cardiovascular risk factors in the three cohorts.

Characteristics	Controls *n* = 89	Non-Structural AF *n* = 92	Structural AF *n* = 104
Age, years (SD)	61.79 (7.81)	64.09 (8.87)	60.04 (9.96)
Males (%)	74.2	84.8	76.9
Smokers (%)	66.3	56.5	32.7 *
Diabetes (%)	10.1	3.3	13.4
Dyslipidemia (%)	17.0	26.1	42.3 *
Hypertension (%)	42.5	30.4	42.3
BMI (Kg/m^2^) (SD)	27.54 (4.48)	27.15 (5.70)	27.65 (5.77)

Controls were matched against the atrial fibrillation groups for age, gender, hypertension, and BMI. SD: standard deviation; BMI: body mass index. Qualitative values compared with Fisher’s exact test. Quantitative values compared with the Student’s *t*-test. * *p* < 0.05.

**Table 2:** Characteristics of heart disease in the structural AF group. (More than one type of cardiomyopathy was possible in each patient).

Characteristics	Numbers
Type of Heart Disease	Total *n* (%) (*n* = 104)
**Valvular heart disease**	
- Mitral regurgitation	71 (68.3)
- Mitral stenosis	2 (1.9)
- Aortic regurgitation	29 (27.9)
- Aortic stenosis	4 (3.8)
**Ischemic heart disease**	9 (8.6)
**Other** (HCM, interatrial communication, etc.)	34 (32.7)
**Dilated left atrium** (>20 cm^2^)	61 (58.7)
**Echocardiography values**	
**Average LVEF (%)**	56
**Average left atrial surface, cm^2^ (SD)**	23 (6.9)
**Average left ventricular ejection fraction, % (SD)**	56.2 (10.1)

HCM: hypertrophic cardiomyopathy, LVEF: Left Ventricular Ejection Fraction, SD: standard deviation.

**Table 3:** Allele distributions of the Cx40 (*GJA5*) and Cx37 (*GJA4*) polymorphisms in the control, non-structural AF, and structural AF groups. Proportions were compared with Fisher’s exact test.

*Genotype*	*Gene*				*p Value vs. Control Group*
***Cx40 −44G > A***	***GG (n, %)***	***AG (n, %)***	***AA (n, %)***	***Total***	
*Control*	*60 (67.4)*	*25 (28.1)*	*4 (4.5)*	*89*	
*Non-Structural AF*	*59 (64.1)*	*27 (29.3)*	*6 (6.5)*	*92*	*p = 0.846*
*Structural AF*	*55 (52.9)*	*41 (39.5)*	*8 (7.7)*	*104*	*p = 0.132*
***Cx40 +71A > G***	***AA (n, %)***	***AG (n, %)***	***GG (n, %)***	***Total***	
*Control*	*60 (67.4)*	*25 (28.1)*	*4 (4.5)*	*89*	
*Non-Structural AF*	*61 (66.3)*	*26 (28.3)*	*5 (5.4)*	*92*	*p = 1.000*
*Structural AF*	*55 (52.9)*	*41 (39.4)*	*8 (7.7)*	*104*	*p = 0.133*
***Cx40* − *26G* > *A***	***GG (n, %)***	***AG (n, %)***	***AA (n, %)***	***Total***	
*Control*	*23 (25.8)*	*42 (47.2)*	*24 (27.0)*	*89*	
*Non-Structural AF*	*26 (28.3)*	*48 (52.2)*	*18 (19.6)*	*92*	*p = 0.511*
*Structural AF*	*27 (26.0)*	*64 (61.5)*	*13 (12.5)*	*104*	***p = 0.029***
***Cx37 1019C > T***	***CC (n, %)***	***CT (n, %)***	***TT (n, %)***	***Total***	
*Control*	*38 (42.7)*	*31 (34.8)*	*20 (22.5)*	*89*	
*Non-Structural AF*	*43 (46.7)*	*41 (44.6)*	*8 (8.7)*	*92*	***p = 0.034***
*Structural AF*	*50 (48.1)*	*43 (41.3)*	*11 (10.6)*	*104*	*p = 0.080*

In bold the genes and genotypes.

**Table 4:** Inclusion and exclusion criteria for the non-structural and structural atrial fibrillation and control groups.

Criteria	Non-Structural AF	Structural AF	Control Group
**Inclusion criteria**	Age > 18 years Paroxysmal or persistent AF Failure of at least 1 anti-arrhythmic drug of class I or III	Age > 18 years Any valvulopathy Left ventricular or atrial dilatation Documented ischemic heart disease Hypertrophic cardiomyopathy Any structural disease	Age > 18 years
**Exclusion criteria**	Reversible AF Clinical heart failure Decrease left ejection fraction Any valvulopathy		History or documented AF Any heart disease including coronary heart disease or significant coronary lesion on angiography Any valvulopathy

AF: atrial fibrillation; NYHA: New York Heart Association heart failure classification.
